# Innovative cryogenic cooling material using spin frustration from abundant elements

**DOI:** 10.1038/s41598-025-29709-5

**Published:** 2025-11-28

**Authors:** Noriki Terada, Hiroaki Mamiya, Akiko T. Saito, Shinji Masuyama

**Affiliations:** 1 National Institute for Materials Science (NIMS), Sengen 1-2-1, Tsukuba, 305-0047 Ibaraki Japan; 2 National Institute of Technology, Oshima College (NIT, Oshima College), Suo-Oshima, Yamaguchi, 742-2193 Japan

**Keywords:** Energy science and technology, Materials science, Physics

## Abstract

Cryogenic cooling technology is essential for modern applications, such as magnetic resonance imaging and quantum computing; however, it currently relies heavily on critical resources such as helium and heavy rare-earth elements. As demand for cryogenic cooling increases, developing alternative technologies that reduce reliance on these scarce resources is crucial. This study introduces regenerator materials from abundant elements—copper, iron, and aluminum—that function as Gifford–McMahon (GM) cryocoolers. These materials achieve cryogenic cooling through the spin frustration effect, where competing magnetic interactions enhance magnetic heat capacity. CuFe₁₋ₓAlₓO₂ demonstrates effective cooling capacity at the helium condensation temperature comparable with that of conventional heavy rare-earth-based materials and surpasses the performance specifications of commercial GM cryocoolers. These findings demonstrate the potential of non-rare-earth magnetic materials for sustainable cryogenic technology, reducing dependence on critical resources.

**Supplementary Information:**

The online version contains supplementary material available at 10.1038/s41598-025-29709-5.

## Introduction

Cryogenic cooling technology, which enables temperatures below 4 K, is primarily used for superconducting electromagnet cooling in medical magnetic resonance imaging (MRI) devices, fundamental physics research, and space engineering^1^. Liquid helium, a key resource for achieving such low temperatures, is a byproduct of natural gas. However, its supply has become increasingly unstable owing to transportation challenges and geopolitical uncertainties^2,3^. Furthermore, as the global shift towards renewable energy reduces natural gas demand, helium production is expected to decline sharply, leading to higher prices and potential supply shortages. To address these issues, cryogen-free refrigeration systems, such as Gifford–McMahon (GM) cycle cryocoolers, have been developed for superconducting electromagnet cooling in MRIs. Unlike systems reliant on liquid helium^4^, GM cryocoolers utilize solid cold storage materials—known as regenerator materials—as their core component. The most common regenerator material, HoCu₂, contains heavy rare-earth elements^5–8^. However, replacing approximately 100,000 operational MRI units with GM cryocoolers would require approximately 100 tons of Ho, far exceeding the global annual production of 10 tons^9^. Beyond MRI applications, cryogenic cooling is vital for cooling quantum devices used in quantum computing^10^, wherein the demand for GM cryocoolers is expected to rise significantly. These trends underscore the urgent need to develop alternative regenerator materials composed of abundant elements, thereby reducing dependence on scarce heavy rare-earth resources.

Regenerators, essential components of cryocoolers, store refrigerant heat at low temperatures during compression-expansion cycles^11^. The selection of regenerator materials depends on the target operating temperature. For temperatures above 20 K, the lattice-specific heat of metals or alloys suffices. However, below 10 K, most materials exhibit negligible lattice-specific heat, rendering them ineffective as regenerator materials. Instead, magnetic materials with high spin-specific heat are used.

**Fig. 1:**
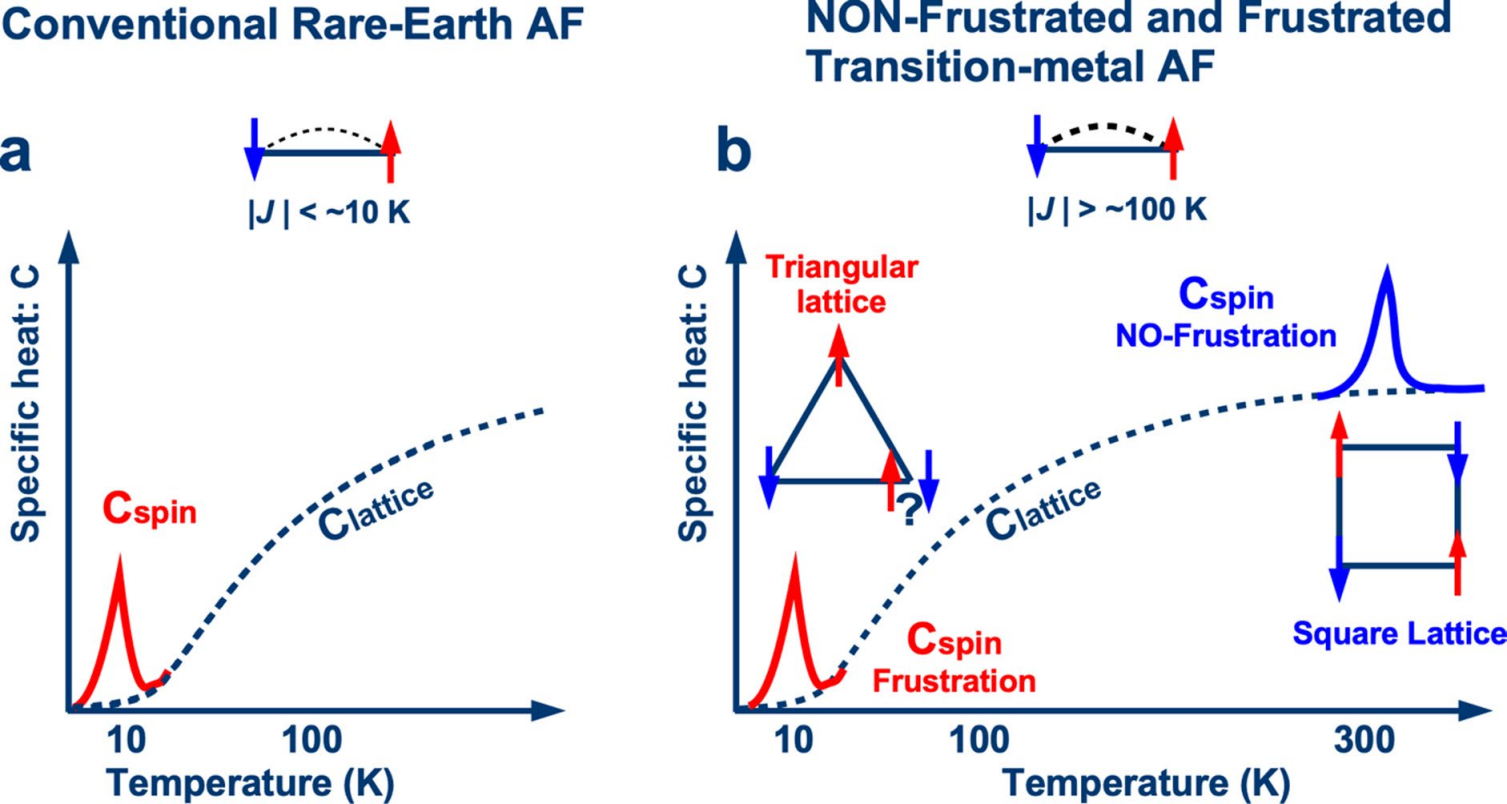
Comparison of conventional and frustration-based systems. Schematic of the temperature dependence of specific heat in: (**a**) conventional rare-earth antiferromagnet (AF) with exchange interaction *J* < ~ 10 K and (**b**) transition-metal AF with *J* > ~ 100 K for non-frustrated (square lattice) and frustrated (triangular lattice) systems.

Traditionally, magnetic regenerator materials for cryogenic applications have relied on compounds containing heavy rare-earth ions^5–8^. These ions exhibit large magnetic moments, attributed to their high total angular momentum quantum number *J*. The total magnetic entropy, *S*_*M*_ $\:=\int\:C/T\:dT$, which influences specific heat, is proportional to ln(2*J* + 1). Heavy rare-earth ions such as Ho^3+^ and Er^3+^ exhibit substantial magnetic moments of 7–8 µ_B_ (Bohr magneton) at cryogenic temperatures. Additionally, the weak magnetic exchange interactions between rare-earth ions result in magnetic phase transitions below ~ 10 K in Ho- and Er-based intermetallic compounds, such as HoCu_2_^12^, Er_3_Ni^13^ and Er(Ni_0.075_Co_0.925_)_2_^14^. These phase transitions induce significant specific heat near the transition temperatures (Fig. 1a).

Conversely, transition-metal compounds face limitations despite containing ions such as Fe^3+^ and Mn^2+^, which have a maximum magnetic moment of 5 µ_B_ owing to their half-filled 3*d* orbitals. While these magnetic moments are adequate for generating high specific heat, the spatially distributed nature of 3*d* orbitals results in stronger exchange interactions than those of the 4*f* orbitals of rare-earth ions. These interactions, typically on the order of several hundred kelvins^15,16^, cause the magnetic phase transition temperatures of many transition-metal compounds to exceed room temperature. Consequently, they become unsuitable for achieving a significant magnetic-specific heat at cryogenic temperatures.

However, leveraging exchange interaction competitions, known as spin frustration in statistical mechanics since the 1950 s, significantly lowers the magnetic phase transition temperature^17^. As illustrated in Fig. 1b, when antiferromagnetic (AF) exchange interactions between neighboring spins occur at energy scales of several hundred kelvins, the AF phase transition in a square lattice (without spin frustration) occurs at a similar temperature. However, in a triangular lattice, where antiparallel spins align at two of the three triangular lattice sites, the third spin remains undecided owing to equivalent energy configurations for up and down orientations. This spin frustration suppresses magnetic ordering, reducing the AF phase transition temperature to the cryogenic range. By exploiting this effect, transition-metal compounds serve as regenerator materials for cryogenic applications.

In this study, we identified CuFeO_2_, an ideal triangular lattice AF compound, with the spin frustration effect, demonstrating high specific heat below 15 K. Using this compound, we successfully developed a regenerator material that achieves a cooling capacity at liquid helium temperatures comparable to that of conventional heavy rare-earth materials. This breakthrough represents a rare-earth-free magnetic regenerator material capable of cooling below the liquid helium transition temperature. Furthermore, this material comprises abundant elements—copper, iron, and aluminum—making it a promising solution for sustainable and environmentally friendly cryogenic cooling technologies.

Additionally, the study investigated the cooling performance and capacity of this rare-earth-free regenerator material in comparison to that of conventional materials under conditions simulating those of operational cryocoolers.

## Results and discussion

### Magnetic specific heat and magnetism

**Fig. 2:**
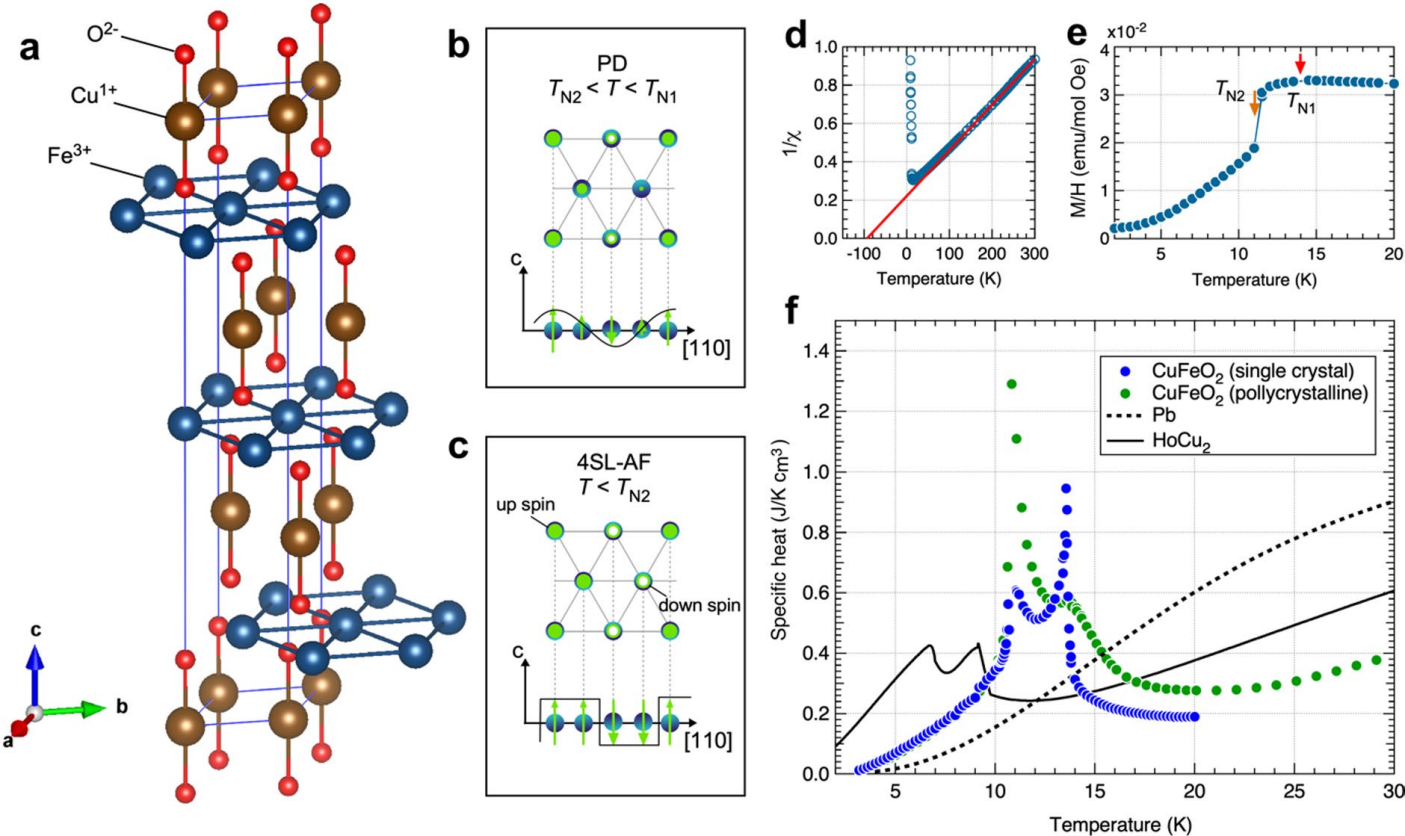
Crystal and magnetic structures, and magnetic and calorimetric properties of CuFeO_2_. (**a**) Crystal structure of CuFeO_2_, showing triangular lattice layers constructed by magnetic Fe^3+^ and separated by nonmagnetic Cu^1+^ and O^2−^. Magnetic structures of the (**b**) PD state and (**c**) 4SL AF state. (**d**) Temperature dependence of inverse magnetic susceptibility. (**e**) Magnetic susceptibility of single-crystal CuFeO_2_ measured under a 100 Oe applied field along the hexagonal c-axis. (**f**) Temperature dependence of specific heat, comparing single-crystal and polycrystalline CuFeO_2_ with HoCu_2_and Pb. (taken from Ref^22^.) contains the data for single-crystal CuFeO_2_.

CuFeO_2_ is a well-known triangular lattice AF^18^ with a delafossite crystal structure exhibiting rhombohedral symmetry in the *R-3 m* space group (Fig. 2a). In this structure, layers of magnetic Fe^3+^ ions form triangular lattices separated by the nonmagnetic Cu^1+^ and O^2−^ ions. The Fe^3+^ ions have a half-filled 3*d*^5^ electronic configuration, yielding a total spin quantum number *S* = 5/2 and a potential magnetic moment of 5 µ_B_ per ion. Magnetic susceptibility measurements reveal a Weiss temperature Q of ~ 95 K (Fig. 2d), indicating an average AF exchange interaction of ~ 95 K. (The Weiss temperature was determined to be approximately 95 K by fitting the high-temperature magnetic susceptibility using the Curie-Weiss law.) However, owing to the spin frustration effect, AF phase transitions occur at much lower temperatures of 14 K (= *T*_N1_) and 11 K (= *T*_N2_) (Fig. 2e). At *T*_N1_, a second-order phase transition occurs from the paramagnetic to the partially disordered (PD) phase, characterized by a spatially sinusoidal spin modulation (Fig. 2b)^19^. This phase transition is marked by a sharp lambda-type peak in the specific heat curve of single-crystal CuFeO_2_, which is typical of second-order transitions^20^ (Fig. 2f). Below *T*_N2_ = 11 K, the magnetic ordering changes to a commensurate (CM) phase, with an up-up-down-down spin arrangement (four-sublattice (4SL) AF state) and fully ordered magnetic moments (Fig. 2c). The specific heat measurement at *T*_N2_ = 11 K shows a high latent heat, indicating a first-order phase transition from the incommensurate to the CM phase^21^.

The specific heat of single-crystal CuFeO_2_ below 14 K is significantly higher than that of Pb, which has the highest lattice-specific heat among metals in this temperature range (Fig. 2f). Furthermore, CuFeO_2_ outperforms the commercially used HoCu_2_ material in specific heat between 9 and 14 K. However, its sharp, peak-specific heat behavior is unsuitable for practical applications, as regenerator materials must continuously retain quantity of heat across a finite temperature range to function effectively in refrigerators^23^.

To mitigate the sharp peak behavior observed around phase transitions in single-crystal CuFeO_2_, we prepared polycrystalline samples of the same composition with slight Al^3+^ substitution at Fe^3+^ sites. Previous studies on the specific heat of polycrystalline CuFeO_2_^24^ have shown that the sharp peak at *T*_*N1*_ is completely broadened, although the peak at *T*_*N2*_ remains prominent (Fig. 2f).

**Fig. 3:**
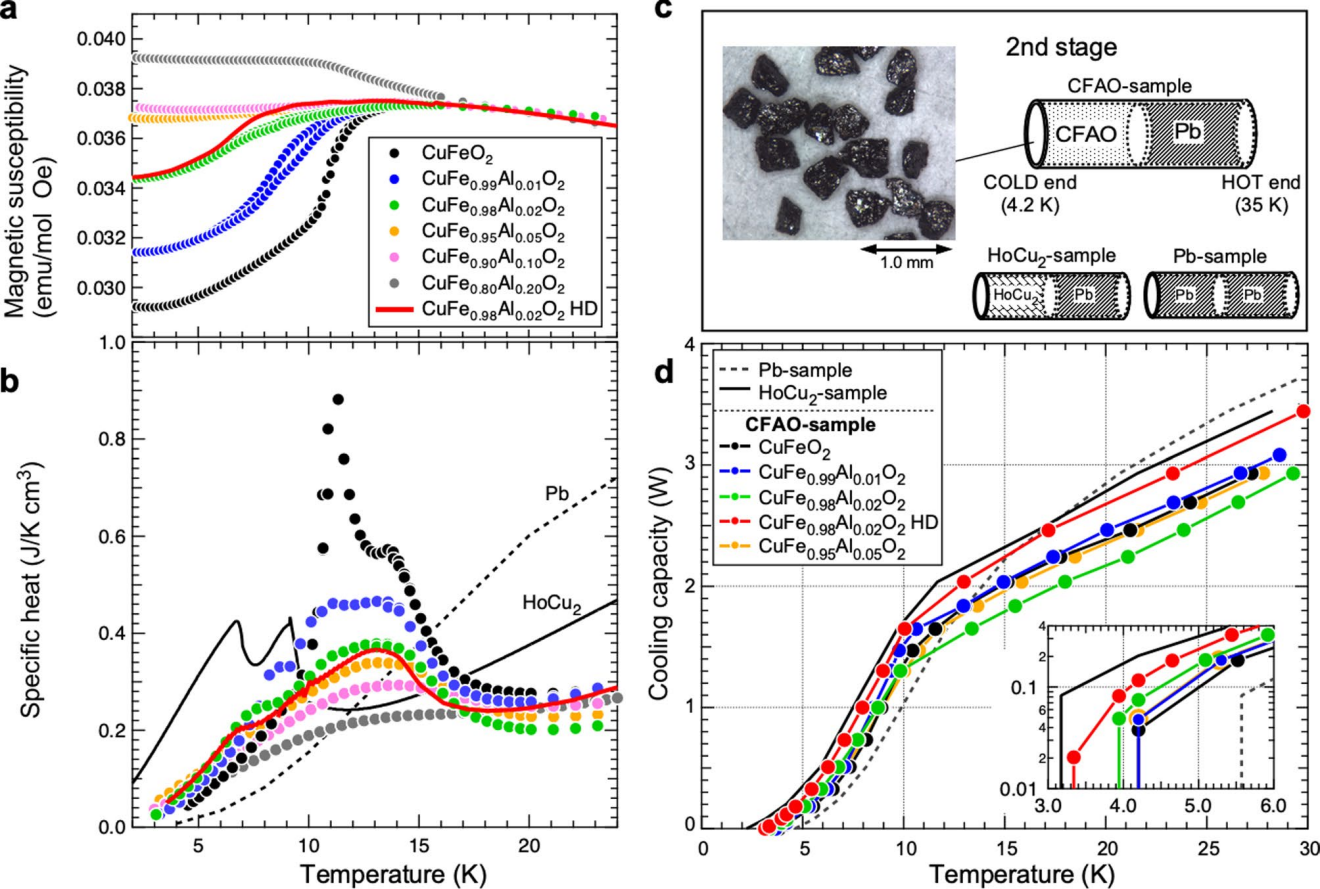
Magnetic susceptibility, specific heat, and cooling capacity of CFAO. Temperature dependence of (**a**) magnetic susceptibility and (**b**) specific heat of powdered CFAO with different x-compositions. (**c**) Photograph of a typical CFAO sample with particle size of 200–500 μm, and schematic of the examined samples (Pb, HoCu_2_, and CFAO). (d) Temperature dependence of cooling capacity of CFAO samples with x = 0.00, 0.01, 0.02, and 0.05. The inset shows a magnification below 10 K on a logarithmic scale. For x = 0.02, we plotted the data for an extra-annealed high-density (HD) sample (CuFe_0.98_Al_0.02_O_2_ HD).

For the chemically substituted polycrystalline CuFe_1 − x_Al_x_O_2_ (CFAO) samples, both *T*_*N1*_ and *T*_*N2*_ peaks were completely broadened (Fig. 3b). Notably, the specific heat below 10 K for samples with *x* $\:\ge\:$ 0.01 exceeded that of both single-crystal and polycrystalline CuFeO_2_. Particularly, the *x* = 0.02 sample exhibited almost twice the specific heat of CuFeO_2_ at 4.2 K, as seen in the Al-concentration dependence of specific heat shown in Supplementary Fig. 3a. This substantial enhancement in specific heat below 10 K is attributed to a magnetic phase transition from the collinear up-up-down-down state to a helical state^25^, as evidenced by a drastic change in magnetic susceptibility (Fig. 3a). This transition, previously reported in CFAO, confirms a fundamental alteration in the low-temperature magnetic structure, leading to distinct specific heat behaviors^26^. Additionally, all CFAO samples showed negligible ferromagnetic components, even under external magnetic fields of several teslas (e.g., *M* = 0.01 T at 1 T; Supplementary Fig. 4). This property is particularly advantageous for practical applications in MRI, as mentioned in detail later.

### Cooling capacity

We prepared CFAO samples with sizes ranging from 200 to 500 μm for compositions x = 0.00, 0.01, 0.02, and 0.05. Additionally, we prepared an extra-annealed sample for x = 0.02 composition by using sintering at 1120 °C, resulting in higher material density. The cooling performance was evaluated using a GM cryocooler (Fig. 3c). These particles were packed into the colder 50% section of the regenerator container at the second stage of the GM cryocooler, with the remaining 50% filled with Pb (Fig. 3c). For comparison, we also prepared HoCu_2_ samples (comprising 50% particles of size 200–300 μm and 50% Pb) and Pb-only samples. The results of the temperature dependence of cooling capacity are shown in Fig. 3d. (The Al-concentration dependence of the cooling capacity at 4.2 K is also shown in Supplementary Fig. 3b.) Notably, CFAO samples exhibited cooling capacities comparable to those of the commercially used HoCu_2_ regenerator materials at ~ 10 K and significantly outperformed Pb-only samples below 14 K. Furthermore, CFAO samples achieved cooling below the helium condensation temperature. Specifically, the extra-annealed x = 0.02 sample reached a minimum temperature of 3.13 K and delivered a cooling capacity of 0.117 W at 4.2 K (inset of Fig. 3d), surpassing the specification value of commercial GM cryocoolers^27^. This represents the demonstration of a rare-earth-free magnetic regenerator material achieving temperatures below the helium condensation point.

### Prospects for practical applications

The CFAO regenerator material shows promising potential for further enhancement. Its current cooling capacity (0.117 W at 4.2 K) already meets the specifications of the commercial GM cryocoolers (0.1 W at 4.2 K). However, improving the filling ratio, currently at 55%, can enhance cooling performance. Spherical granulation processes increase the filling ratio to ~ 65%, bringing CFAO closer to optimal performance.

The relationship between particle size, which determines the pressure loss across the helium gas, and thermal conductivity is also an important parameter that determines the cooling capacity in GM cryocoolers^28^. The intrinsic thermal conductivity data of CuFeO_2 _and some doing systems, which were measured with the single crystal samples, have been reported in previous studies^29,30^. According to these studies, the thermal conductivity averaged over two orthogonal directions parallel and perpendicular to the hexagonal c-axis in CuFeO_2_ has approximately 1 W/m K for the temperature range 4–10 K. We measured the thermal conductivity for the polycrystalline sample of CFAO. (Supplementary Fig. 5**)**. The value is almost one order magnitude lower than the single crystal value. It is considered to be caused by the effect of grain boundaries in the polycrystalline sample measured. The commercially used HoCu_2_ has a similar thermal conductivity value (0.28 W/m K for 4 K < *T* < 20 K)^31^ to the polycrystalline sample of CFAO. Therefore, the optimum particle size is expected to be around 200–500 μm as expected from HoCu_2_ particles, but further improvement in cooling capacity can be expected by fine tuning the particle size.

Further improvements can be achieved by combining CFAO with other materials. While CFAO exhibits a relatively high specific heat above 5 K, comparable to that of HoCu_2_, its specific heat decreases significantly below this temperature, limiting its cooling capacity to 4.2 K. In order to improve the weakness of the specific heat below 5 K in CFAO, another material that shows high specific heat below 5 K can be additionally used. For example, for previous rare earth regenerator study, Gd_2_O_2_S material with a large specific heat around 4 K is used in combination with HoCu_2_, leading to improvement of the cooling capacity at 4.2 K^22,23^. Based on these previous studies^22,23^, we anticipate that combining CFAO with other frustrated materials with high specific heats around 4.2 K could address this limitation.

CFAO also presents magnetic advantages for applications such as MRI superconducting magnet cooling. Cryogenic GM refrigerators are placed in high magnetic field environments during the operation of the superconducting electromagnets in MRI devices. In such conditions, the magnetic force between ferromagnetic regenerator materials in the refrigerator and the magnetic field generated by the superconducting magnet is often problematic. Magnetic forces may pull components of the GM refrigeration unit, potentially causing uneven wear, deformation, or damage. This could result in the refrigeration unit failing to operate correctly. However, CFAO is an AF with weak field-induced ferromagnetic component, even under external fields of several teslas (e.g., *M* = 0.01 T at 1 T; Supplementary Fig. 4) at 4 K. Therefore, in contrast to commercially used HoCu_2,_which exhibits high field-induced magnetization, the CFAO regenerator is unlikely to experience significant magnetic forces during operation. Evern better, the specific heat for CFAO remains almost unchanged when applying magnetic even up to 12 T^32^. Moreover, its low magnetization offers the added advantage of minimizing magnetic noise, a problem for MRI operations with an alternating current field, owing to particle vibration caused by the refrigerator cycle. These advantages make the CFAO material suitable for use in GM refrigerators for MRI.

**Fig. 4:**
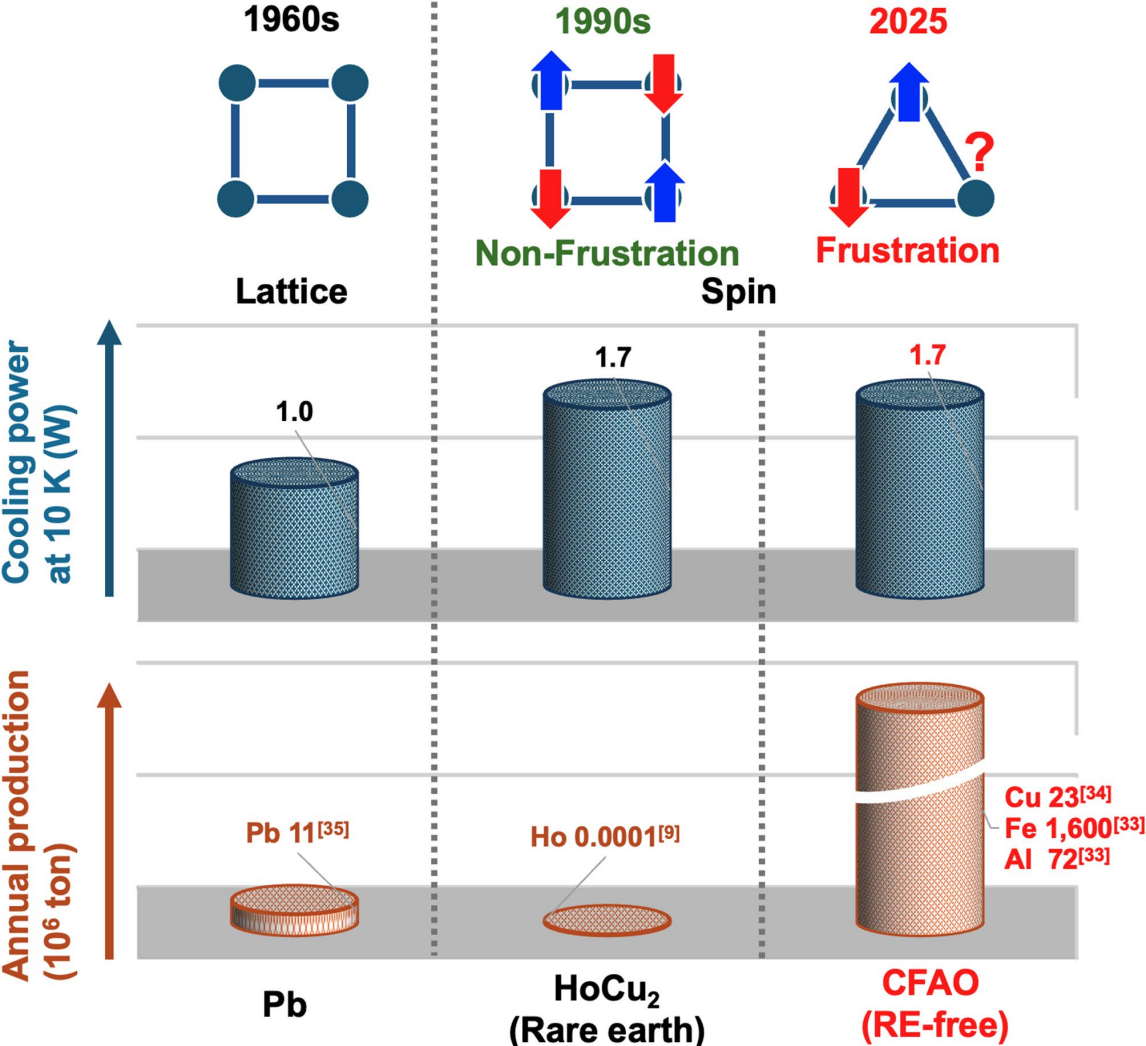
In the 1960’s, GM refrigerators using Pb cryostats began to be used, and in the 1990’s, the heavy rare earth magnetic cryostat HoCu_2_was developed and has been used for the next 30 years. The frustrated CFAO developed in this study enables the same level of cryogenic cooling without using of rare earth elements. The values of annual production were taken from the references^9, 33–35^.

In conclusion, magnetic regenerator materials containing heavy rare earths were developed in the 1990’s to replace Pb used since the 1960’s, and have been used as an alternative cooling technology to liquid helium for more than 30 years.(Fig. 4) However, there are concerns about the supply of resources of rare-earth elements to meet the recent increase in demand for cryogenic cooling technology. In this study, we demonstrated that CFAO, a rare-earth-free regenerator material, achieved a cooling capacity exceeding commercial GM cryocooler specifications at the helium condensation temperature. This was enabled by leveraging the spin frustration effect, which suppresses magnetic phase transitions to cryogenic temperatures despite strong exchange interactions. These findings introduce a viable alternative to conventional regenerator materials and highlight the potential of frustrated magnets in advancing environmentally sustainable cryogenic cooling technologies.

## Methods

*Sample Preparation*: Polycrystalline CFAO powder samples were prepared via solid-state reaction. Pelletized powders of Cu_2_O (> 99.9%), a-Fe_2_O_3_ (> 99.9%), and a-Al_2_O_3_ (> 99.9%) were mixed in a molar ratio of 1:1-x: x. The mixtures were heated at 1050 °C for 2 d in an Ar atmosphere, with heating and cooling rates of ~ 1 °C min^−1^ and ~ 3 °C min^−1^, respectively. X-ray diffraction (RIGAKU MiniFlex Cr-target) confirmed the single-phase nature of all synthesized samples (Supplementary Fig. 1). The pelletized samples were granulated using a tungsten mortar and separated into particle sizes between 200 and 500 μm. The particle density was 88%, and the fill rate was 55% (Supplementary Fig. 2a). For the x = 0.02 composition, an additional sample was prepared with extra annealing at 1120 °C for 2 d under identical conditions (Supplementary Fig. 2b). HoCu_2_ particles (200–500 μm in diameter) and Pb (210–250 μm in diameter) particles were purchased from Toshiba Materials Co., Ltd. and Shouki Seisakusyo, respectively.

*Specific Heat Measurements*: Specific heat was measured using the relaxation method with a physical property measurement system (PPMS) from Quantum Design (QD).

*Magnetometry Measurements*: Magnetization data were obtained using a QD magnetic property measurement system. The temperature dependence of magnetic susceptibility was measured under a field strength of 100 Oe, while the magnetic field dependence of magnetization was measured at 4 K.

*Scanning Electron Microscopy (SEM)*: SEM imaging was performed using the JEOL JCM-6000PLUS system.

*Cooling Test*: A conventional two-stage GM cryocooler (SHI, RDK101D model) with an air-cooled compressor (1.3 kW, 60 Hz) was used to evaluate the cooling capacity of each regenerator material. The cryocooler operated at a frequency of 1.2 Hz, with helium as the refrigerant at a pressure range of 0.8 to 2.1 MPa. The vacuum insulation chamber was evacuated by the turbo molecular pump, and the pressure was less than 10^-4^ Pa. The regenerator materials were inserted into a cylinder at the second stage, which was divided into two parts halfway along the longitudinal direction (temperature gradient) by a felt sheet and a stainless-steel mesh. The hotter side of the cylinder was filled with spherical Pb particles (210–250 μm in diameter), while the colder side contained CFAO, HoCu_2_ (200 μm in diameter), or Pb particles. The first and second stages covered temperature ranges of 300 to 35 K and 35 to 4 K, respectively. The schematic illustration for the experimental setup was shown in Supplementary Fig. 6. We used the calibrated silicon-diode temperature sensors for the temperature measurements. In order to measure cooling capacity for the cryocooler, we put the electric heaters made of high-nickel alloys on the first and second stages and measure the heater power to determine the cooling capacity at each temperature.

*Thermal conductivity measurement*: For the thermal conductivity measurement we employed the “two thermometer-one heater method” manufactured by the QD’s PPMS . Cernox thermometers monitor the temperature of two polished oxygen-free high-conductivity copper plates fixed to the sample (2.5 x 1.0 x 11 mm^3^) with silver epoxy paste. The gap between the plates was 3.6 mm. The sample prepared for thermal conductivity measurements was annealed at 1120 °C after the solid state reaction at 1050 °C.

## Supplementary Information

Below is the link to the electronic supplementary material.

Supplementary Material 1

## Data Availability

The data supporting the plots within this paper and other study findings are available from the corresponding author upon reasonable request.
