# Supplementary material for: Innovative cryogenic cooling material using spin frustration from abundant elements
Source: Sci Rep. 2025 Nov 28;15:44240. doi: 10.1038/s41598-025-29709-5 (PMC12722759; doi:10.1038/s41598-025-29709-5)
Supplement: Supplementary file 1 — Supplementary Material 1 [file 41598_2025_29709_MOESM1_ESM.docx]

Supporting Information

Innovative Cryogenic Cooling Material Using Spin Frustration from Abundant Elements

*Noriki Terada*, Hiroaki Mamiya, Akiko T. Saito, and Shinji Masuyama*

1. **X-ray diffraction**

Supplementary Fig. 1 X-ray diffraction patterns for all samples that we prepared for the specific heat measurements. For the comparison, the calculated diffraction pattern is also shown.

1. **Scanning Electron Microscope**


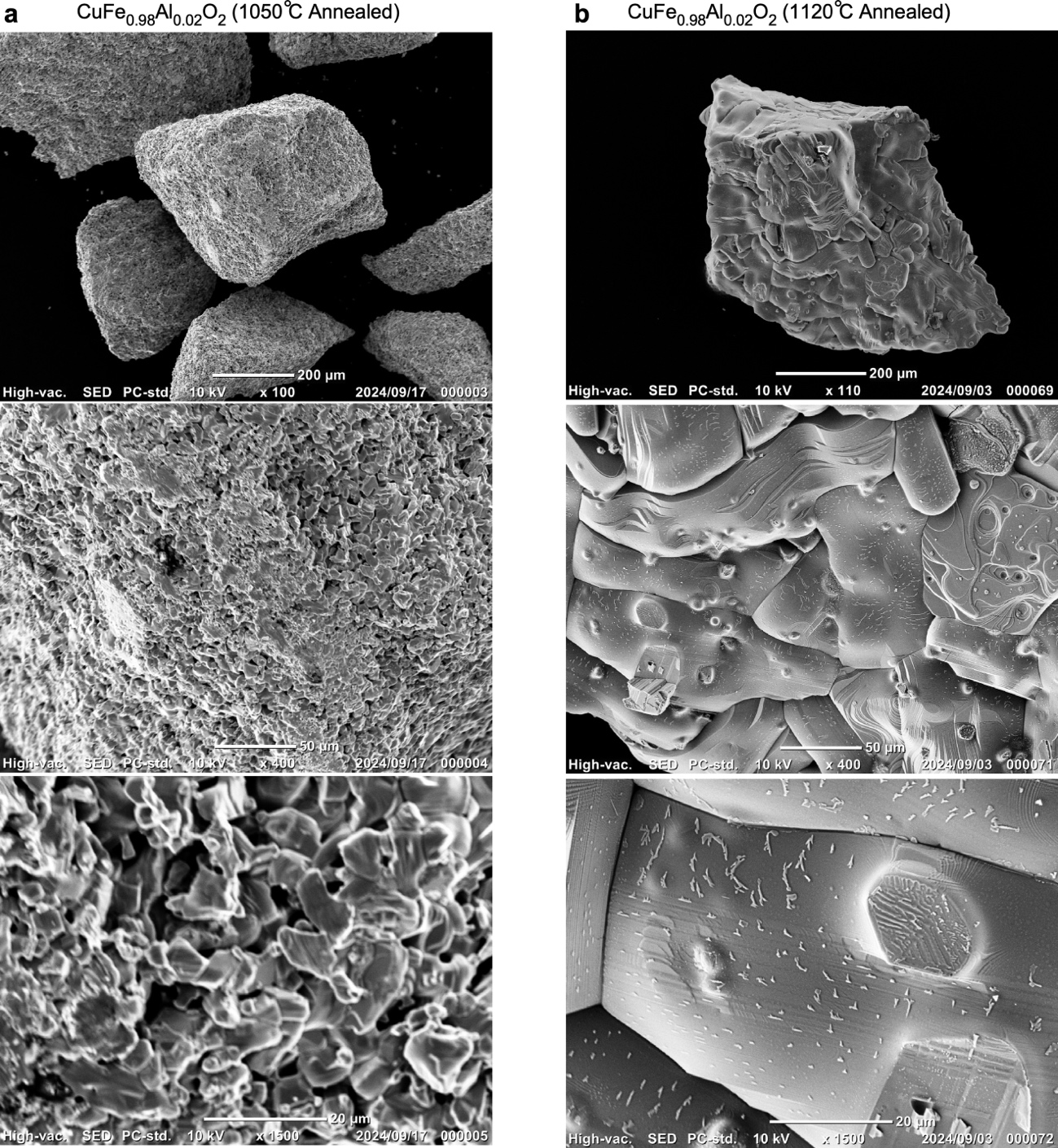


Supplementary Fig. 2 SEM images of typical CFAO (x=0.02 sample) particles after annealed at (a)1050 ºC and (b) 1120 ºC.

1. **Al-concentartion dependence of specific heat and cooling capacity**

**
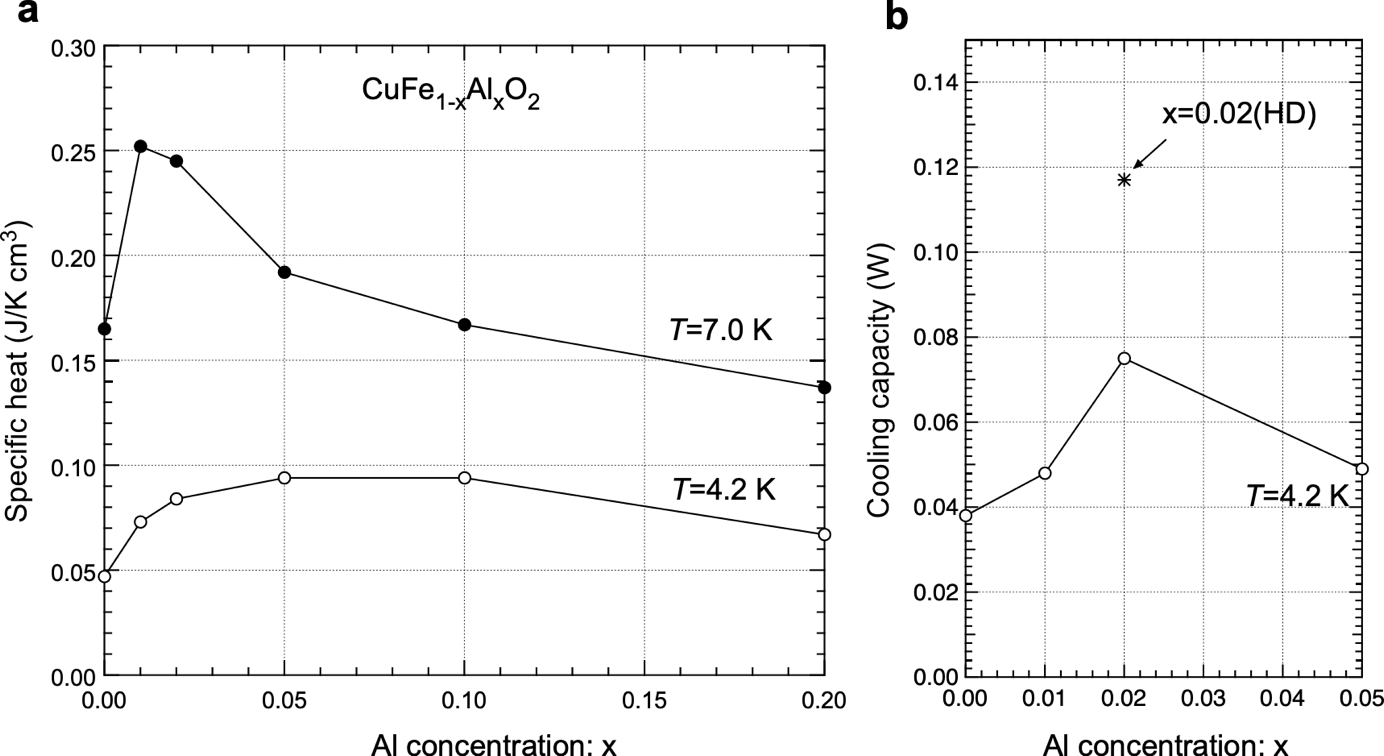
**

Supplementary Fig. 3 Al-concentration dependence of (a) the specific heat at 4.2 K and 7.0 K, and (b) the cooling capacity in CuFe_1-x_Al_x_O_2_ materials. The asterisk symbol in (b) denotes the data for the high density sample of x=0.02.

1. **Magnetization**


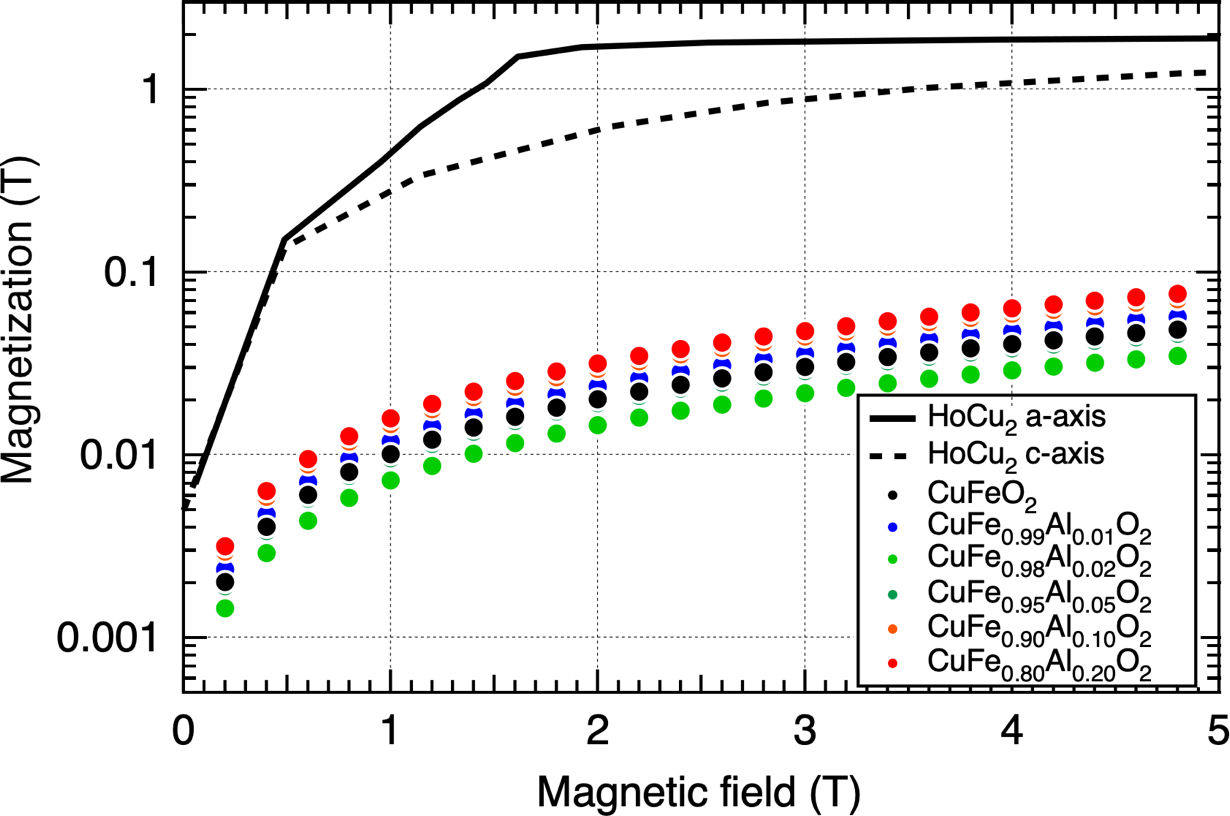


Supplementary Fig. 4 Magnetic field dependence of magnetization for CuFe_1-x_Al_x_O_2_. The data were subtracted by magnetization due to tiny amounts of α-Fe_2_O_3_ impurities in the samples. As the reference, the magnetization curves for commercially used regenerator material HoCu_2_ along two principal directions are plotted. The data of HoCu_2_ were taken from Ref. [12] in the main text.

1. **Thermal conductivity**

Supplementary Fig. 5 Temperature dependence of thermal conductivity for plycrystalline CuFe_0.98_Al_0.02_O_2_.

1. **Experimental setup for measuring the cooling capacity**


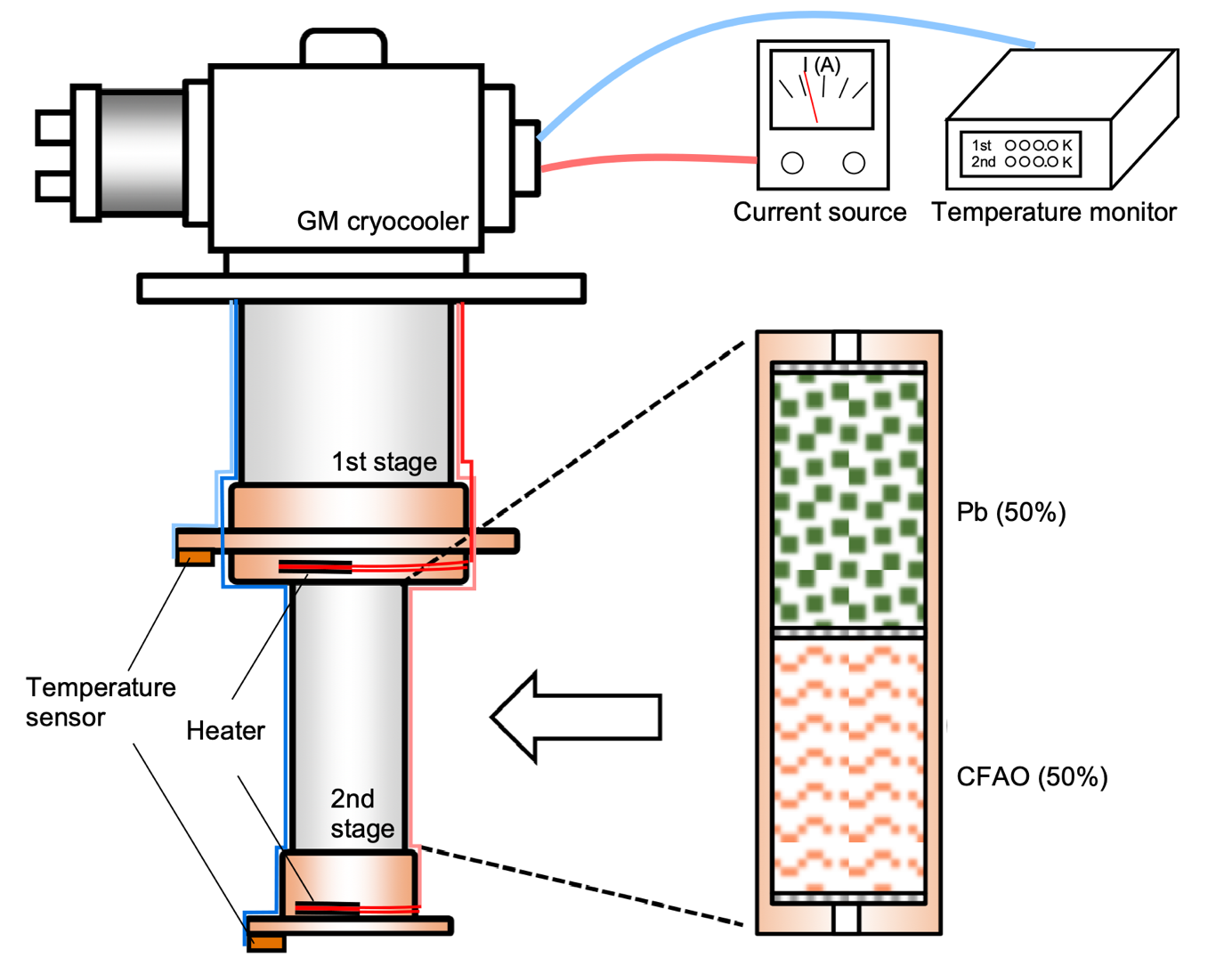


Supplementary Fig. 6 Schematic illustration of the experimental setup for cooling capacity measurements. Two tempearture sensors are placed on first and second stages, and electric heaters are also placed on the cold heads on each stage to measure the cooling capacity.
